# P-648. Significant Increase in Influenza-related Staphylococcus aureus Pneumonia During the 2024-2025 Influenza Season in New York City

**DOI:** 10.1093/ofid/ofaf695.861

**Published:** 2026-01-11

**Authors:** Kimberly Mae D Soultan, Anjali Kewalramani, Alison Bradbury, Yongsheng Wang, Marie Abdallah, Briana Episcopia, John Quale

**Affiliations:** SUNY Downstate Medical Center, Brooklyn, NY; SUNY Downstate Medical Center, Brooklyn, NY; NYC health and hospitals/Kings County, Brooklyn, New York; NYC Health + Hospitals/Kings County, Brooklyn, New York; New York City Health and Hospital/Kings County, Brooklyn, New York; NYC H+H/ Kings County, Garden City, New York; NYC Health + Hospitals/Kings County, Brooklyn, New York

## Abstract

**Background:**

Host responses to infection with influenza A virus (IAV) impair clearance of bacteria in the nose and lungs. Prior studies have emphasized the adverse outcomes due to methicillin-resistant Staphylococcus aureus (MRSA) pneumonia in patients co-infected with IAV.

**Methods:**

Line listings of admitted adult patients, from an 11-hospital safety net healthcare system in New York City, with community-onset pneumonia and positive respiratory cultures for SA were obtained during the height of the 2024-2025 influenza season. Records were reviewed to determine co-infection with IAV and clinical outcomes. Results were compared to the same time period for the 2023-2024 influenza season. Infection rates were determined using the total number of admissions for the hospital system during the specified time period.

**Results:**

From Jan 12-Feb 28, 2024 there were 55 patients admitted with positive community-onset respiratory cultures with SA (2.2 patients per 1000 admissions). During the same time period in 2025, there were 84 such patients (3.2 patients per 1000 admissions, P=.03). Influenza-related cases drove this increase: from 7 cases in 2024 (rate 0.28 cases/1000 admissions) to 28 cases in 2025 (1.1 cases/1000 admissions, P=.0005). Of the 28 influenza-related cases in 2025, 27 had co-infection with IAV. All of the 28 patients admitted influenza and positive respiratory cultures for SA had radiographic evidence of pneumonia; 15 had methicillin-susceptible SA (MSSA) and 13 had MRSA. Of the 28 patients, 13 had concomitant bacteremia, 7 required ICU admission, and 6 died. Only two of the 28 patients received the seasonal influenza vaccination before admission.

**Conclusion:**

During the peak of the 2024-2025 influenza season in New York City, there was a significant rise, compared to the 2023-2024 season, of admitted patients with SA pneumonia co-infected with IAV. Co-infection with IAV was not restricted to MRSA, as over half had MSSA. Nearly half had concomitant bacteremia, and the mortality rate was 21%.

**Disclosures:**

All Authors: No reported disclosures

